# Introducing the Naturalistic Expression Labeling Task (NELT): Associations with posed expression labeling, empathy, and general cognitive ability

**DOI:** 10.3758/s13428-026-02944-y

**Published:** 2026-04-13

**Authors:** Louisa A. Talipski, Romina Palermo, Clare A. M. Sutherland, Gilles E. Gignac, Linda Jeffery, Kate Crookes, Jeremy B. Wilmer, Eva G. Krumhuber, Jason Bell, Amy Dawel

**Affiliations:** 1 School of Psychological Science, The University of Western Australia, Crawley, Australia; 2 School of Medicine and Psychology, The Australian National University, Canberra, Australia; 3 School of Psychology, King’s College, University of Aberdeen, Aberdeen, Scotland; 4 School of Population Health, Curtin University, Bentley, Australia; 5 Department of Psychology, Wellesley College, Wellesley, USA; 6 Department of Experimental Psychology, University College London, London, UK

**Keywords:** Expression labeling, Naturalistic, Test development, Empathy, Cognitive ability

## Abstract

As decoding emotional expressions is essential to navigating the social world, it is imperative that measures of facial expression labeling ability are psychometrically rigorous and easy to administer. Unfortunately, most studies have used images of prototypically posed expressions, which lack the nuance and variation of real-life expressions and are often perceived as fake. To address the need for a more ecologically valid measure of expression labeling, we introduce the Naturalistic Expression Labeling Task (NELT), modeled after an established posed expression labeling task. We investigated whether the NELT shows expected associations with empathy and general cognitive ability, and the extent to which these associations align with those found for the corresponding posed expression task, despite differences in the realism and perceived genuineness of the expressions. Across three studies, we found that the NELT had strong psychometric properties—including high reliability—that make it well suited to examining individual differences in expression labeling ability. While both the NELT and the posed expression task showed similarly sized positive associations with measures of cognitive and affective empathy, the NELT exhibited a stronger positive association with cognitive ability than did the posed expression task. Our findings suggest that naturalistic expressions can provide insights into expression labeling ability that are at least as robust as those derived from posed expressions. The NELT can serve as a valuable tool for researchers seeking to enhance the ecological validity of their studies by incorporating naturalistic stimuli.

Reading facial expressions is critical to effective social functioning, underscoring the need for psychometrically rigorous tests of this ability. Most studies of facial expression perception have relied on stimuli posed under highly controlled conditions, with consistent lighting and a limited number of viewpoints (Dawel et al., 2017, 2022). Capturing these expressions typically involves instructing people (often actors) to display a given emotion (e.g., Lundqvist et al., 1998) or to move certain facial muscles believed to signal specific emotions (e.g., Ekman & Friesen, 1976; Langner et al., 2010). The prevalence of posed expressions in emotion research is problematic because such expressions are (deliberately) highly prototypical and fail to capture the nuanced and varied repertoire of real-life expressions (Barrett et al., 2019; Dawel et al., 2025). Moreover, many popular posed expression stimuli are perceived by participants as faking emotion (Dawel et al., 2017, 2025), and research has found different results when naturalistic and spontaneous expressions are used instead (Bothe et al., 2024; Cong et al., 2025; Davis & Gibson, 2000; Dawel et al., 2019a, 2019b; Gunnery & Ruben, 2016; Krumhuber et al., 2021; LaRusso, 1978). Thus, there are compelling reasons to use naturalistic expressions—that is, expressions from uncontrolled, real-world settings—which are more reflective of daily experiences and may foster greater participant engagement. However, despite growing recognition of the importance of ecological validity in face perception research (e.g., Barrett et al., 2019; Krumhuber et al., 2017; Long et al., 2023; Pasqualette et al., 2023; Sutherland et al., 2017), there is a lack of psychometrically validated measures of naturalistic expression labeling ability. As such, there are a limited number of studies examining individual differences in this ability, and a lack of clarity as to whether findings obtained from posed expressions generalize to naturalistic expressions.

Here, we address this gap by developing a novel test of naturalistic expression labeling ability and investigating its associations with an analogous measure of posed expression labeling ability (Palermo et al., 2013, 2018), empathy, and cognitive ability. While past literature often refers to expression *recognition* ability, task accuracy is typically determined by alignment between the emotion label a participant selects and a consensus or database-provided label for a stimulus, rather than alignment with the emotion felt by the expresser (which may be absent for posed expressions and difficult to ascertain for naturalistic expressions). This common method of measuring accuracy means that many published “expression recognition” tasks can be more accurately described as measures of expression labeling ability. Thus, we prefer the terms “expression labeling” and “expression perception,” and avoid the term “expression recognition.”

## Why naturalistic expressions?

Here, we define naturalistic expressions as those recorded as they naturally occur in uncontrolled, real-world settings. Naturalistic expressions may include both spontaneous (or involuntary) expressions, which reflect natural reactions to emotional events, and voluntary natural expressions, such as smiling for non-emotional reasons (e.g., to be polite). A key advantage of naturalistic expressions is that, unlike expressions deliberately posed by actors, they are the types of facial displays people encounter in their everyday interactions, which assists with engaging participants in experimental tasks. Because they are not controlled, naturalistic expressions also include substantial variation in viewpoint, eye gaze, and lighting, which are often missing from lab-based stimuli. Finally, and most critically, naturalistic facial behavior differs in important ways from that seen in posed expressions, and as we discuss below, these physical differences in facial appearance can lead to differences in participant responses. Physically, posed expressions are highly prototypical and often exaggerated, such that deciphering these expressions is like reading a “human emoticon.” In contrast, naturalistic expressions tend to be more nuanced and ambiguous in their emotional meaning (Cohn & Schmidt, 2004; de la Harpe et al., 2024; Küster et al., 2022), which means they may require a greater level of skill to interpret. Accordingly, tests of naturalistic expression perception may reveal deficits in real-life skills that are not observable with posed expressions. While naturalistic expressions may be perceived as conveying genuine feelings of emotion, some may not. Traditional posed expression stimuli, however, are often perceived as less genuine and faking emotion (Dawel et al., 2025; Krumhuber et al., 2021).

There are now numerous studies showing critical divergences in results obtained from naturalistic and spontaneous expressions, or those perceived as showing genuine emotion, compared to results from posed expressions, often perceived as faking emotion.^1^ First, a large body of evidence shows that people respond more positively to smiles that are perceived as genuine relative to those that are not (Bothe et al., 2024; Dawel et al., 2019a; Gunnery & Ruben, 2016; Krumhuber & Kappas, 2022). Second, for labeling specifically, a recent study found that compared to accuracy for posed expressions, accuracy for spontaneous expressions was lower for negative expressions but higher for positive ones (Cong et al., 2025). Studies in the clinical domain also reveal divergences. For example, individuals with schizophrenia demonstrate *superior* expression labeling ability compared to matched control participants when tested with spontaneous emotional expressions, while the opposite pattern is evident when posed expressions are used (Davis & Gibson, 2000; LaRusso, 1978). In another example, a major theory that explains the affective features of psychopathy was supported only when tested with spontaneous emotional expressions and *not* when tested with posed ones (Dawel et al., 2019b). These findings highlight the potential for expressions that are naturally or spontaneously elicited to provide different, and potentially more ecologically valid, insights into important aspects of human behavior.

Assuming robust psychometric properties are maintained, it would therefore be beneficial to include naturalistic stimuli in expression labeling tasks to uncover ecologically valid associations with other critical abilities. To address this gap, the present study introduces the Naturalistic Expression Labeling Task (NELT), comprising images from a new set of naturalistic facial expressions. These images show expressions from a variety of contexts and have been validated to be perceived as expressing genuine emotion (see “Method”). The new task follows the format of an established labeling task containing posed facial expressions (Palermo et al., 2013, 2018). Critically, the original posed task was constructed to provide a reliable and valid measure of individual differences in ability across the general population, with a good range of scores that sits above floor and below ceiling, and high internal reliability (Cronbach’s α = .80 to .86). The posed task has already demonstrated the ability to reveal associations with perceptual mechanisms underlying expression perception (Palermo et al., 2018) and affective measures (e.g., anxiety; Alharbi et al., 2020). Furthermore, the original posed task is sensitive to clinical-level impairments (e.g., traumatic brain injury; Rigon et al., 2018) and autistic-like traits (Bothe et al., 2019).

In the present study, we examined associations between the NELT and other measures targeting cognitive and affective processes. We also included the original posed expression task to determine whether these associations differ across tasks. Examining the relationship between expression labeling ability and other cognitive and affective processes is important because expression perception is influenced not just by the characteristics of the emotional expressions, but also by top-down factors and other individual differences (e.g., Barrett & Kensinger, 2010; Binetti et al., 2022). Moreover, it is possible that these associations could vary depending on whether posed or naturalistic expressions are used. In this paper, we examine associations with empathy and general cognitive ability—two abilities that, like expression labeling ability, are associated with positive life outcomes (e.g., Davis, 2015; Schmidt & Hunter, 2004). Both abilities have also been conceptually linked with expression labeling ability, but empirical evidence of these associations has primarily relied on posed expressions (e.g., Olderbak & Wilhelm, 2017; Schlegel et al., 2020). Below, we describe current understanding of the links between expression labeling and these other abilities.

## Expression labeling ability and individual differences

One key individual difference variable that has been investigated in relation to expression labeling ability is empathy. Acknowledging that definitions of empathy vary (Batson, 2009; Cuff et al., 2016), we define it in general terms as the ability to understand and emotionally respond to how someone else is feeling. From a theoretical perspective, some researchers have argued that accurate perception of another’s emotion is a necessary precursor to the experience of empathy (e.g., Corrigan, 1997; Ochsner, 2008), while others suggest that expression perception ability and empathy are related because both are aspects of “emotional intelligence” (e.g., Petrides, 2009). Past work has also distinguished between cognitive and affective empathy. Cognitive empathy is the ability to construct a working model of another’s emotional state, whereas affective empathy involves being sensitive to and vicariously experiencing another’s emotions (Reniers et al., 2011). Evidence for a dissociation between the two forms of empathy comes from neuroanatomical studies showing the involvement of distinct brain regions for each type (Zaki & Ochsner, 2012) and from behavioral studies with distinct neurocognitive groups (e.g., those showing differences in cognitive but not affective empathy in autism; Mazza et al., 2014; Rueda et al., 2015). Further support comes from a meta-analysis by Olderbak and Wilhelm (2017) showing that the two forms of empathy have less than 25% shared variance, indicating they are largely dissociable.

Despite the clear theoretical links, empirical studies have produced mixed findings regarding the relationship between facial expression labeling ability and cognitive and affective empathy. While some have found that expression labeling ability is associated with both types of empathy (Lockwood et al., 2017; Melchers et al., 2016; Olderbak & Wilhelm, 2017; Schlegel et al., 2019), others found an association only for cognitive (Jiang et al., 2014; Lui et al., 2016; Moret-Tatay et al., 2022; Mullins-Nelson et al., 2006) or affective empathy (Besel & Yuille, 2010; Gery et al., 2009; Riggio et al., 1989). These inconsistent results may be partly due to the reliance on posed expressions. Spontaneous expressions that are perceived as showing genuine emotion elicit greater activity in emotion-related neural regions and the mirror neuron system, which is implicated in empathic responding (McLellan et al., 2012). In addition, facial mimicry, also implicated in empathic responding, is stronger for Duchenne smiles (characterized by eye muscle movement that typically signals genuine enjoyment) than for non-Duchenne smiles (Krumhuber et al., 2014). This pattern suggests that naturalistic expressions may elicit a more embodied response than those of posed expressions. Therefore, using more naturalistic expressions might reveal stronger associations with empathy—particularly affective empathy, which involves an embodied response to others’ emotions (Ferrari & Coudé, 2018).

The inconsistency in past results may also be due to the use of expression labeling measures that are not particularly sensitive to individual differences. For instance, some of the studies (e.g., Besel & Yuille, 2010; Riggio et al., 1989) that investigated the relationship between expression labeling and empathy presented a selection of posed expressions but did not psychometrically validate the tasks. The lack of rationale for image selection, together with limited reporting of the descriptive statistics for the tasks, makes it unclear whether the tasks had adequate internal reliability and/or range in performance levels sufficient to accurately uncover associations with one or both types of empathy (see Goodhew & Edwards, 2019, for a general discussion of such psychometric issues). Given that previous research has focused on expression labeling tasks containing posed facial expressions and/or has used measures that might be insufficiently sensitive to individual differences, one aim of the current study was to advance the field by testing these relationships using validated naturalistic and posed expression labeling tasks that are designed to capture individual differences.

Facial expression perception ability has also been proposed to have a positive relationship with general cognitive ability. Gignac and Szodorai (2024) characterize cognitive ability operationally as an individual's “maximal capacity to complete a novel standardized task with veridical scoring using perceptual/cognitive processes” (p. 3). Given that tests of expression labeling fundamentally align with this definition, a positive correlation with general cognitive ability appears theoretically plausible. In particular, integrating visual cues across the face to recognize an expression requires the ability to reason and problem-solve in real time, as well as—in the case of tasks requiring labeling—access to stored (crystalized; Gc) knowledge to assign an emotion label (Davitz et al., 1964). Given the role of visual perception in facial expression perception, measuring expression labeling ability might also provide a means of measuring “Gv,” the visual intelligence dimension included in the Cattell–Horn–Carroll theory of cognitive abilities, which has received substantial support in the literature (Schneider & McGrew, 2018). Some contemporary researchers (e.g., Wilhelm & Kyllonen, 2021) have even suggested that socioemotional abilities comprise their own intelligence factor, with expression perception a particular type of socioemotional ability (e.g., Meyer et al., 2021; see also Walker et al., 2023, 2025). A large meta-analysis of the relationship between expression perception ability and general cognitive ability supports theoretical arguments for a link between the two constructs, revealing a small-to-moderate correlation of *r* = .19 (Schlegel et al., 2020). However, 97% of the meta-analyzed effect sizes were from studies using posed facial expressions, and of the remaining effect sizes, none came from a study directly comparing naturalistic and posed expressions. Consequently, another aim of our study was to determine whether testing with naturalistic expressions produces the same findings as those obtained with posed expressions, as this helps identify domains in which findings from posed expressions can be generalized to naturalistic expressions. Another consideration is that, given the robust relationship between posed expression perception ability and general cognitive ability (Schlegel et al., 2020), it may be important to control for cognitive ability when examining relationships between expression perception and other variables. Measuring general cognitive ability could therefore facilitate understanding of the relationships between expression perception and empathy.

In a recent study, Scarpazza et al. (2025) examined whether expression labeling ability and the ability to evaluate expression authenticity are related to empathy and general cognitive ability, using a new task (the Emotion Authenticity Recognition task) comprising both “authentic” and posed expressions. Although these authors observed positive associations between expression labeling ability and both empathy and cognitive ability, they did not report these associations separately for authentic and posed expressions, which means it is unknown whether these associations differed by expression type. In addition, the authors did not report the reliability of the task or optimize the task for capturing a range of performance levels (labeling accuracy for each item was at ceiling). Our study addresses these issues by using separate—yet comparable—naturalistic and posed expression labeling tasks that were optimized for individual-differences research, separately assessing their relationships with empathy and general cognitive ability, and reporting their psychometric properties.

Given the inconsistency in past findings, our general hypothesis was that expression labeling ability would be positively associated with empathy, but we did not have specific predictions about whether this association would differ between cognitive and affective empathy, or between naturalistic and posed expression labeling. We also predicted that cognitive ability would be positively related to facial expression labeling in general, but were open as to whether (and how) this relationship might differ according to expression type. One possibility is that identifying naturalistic expressions—which tend to be more nuanced and subtle than posed expressions (Russell, 1994)—relies more on embodied responses than on cognitive ability (Krumhuber et al., 2014; Likowski et al., 2012; Niedenthal et al., 2000), which could result in a smaller association with cognitive ability than seen with posed expressions. Alternatively, the more nuanced nature of naturalistic expressions might make them more cognitively demanding to identify, leading to a stronger association.

## The present research

In the current work, we describe the newly validated NELT, highlighting similarities and differences with our previously developed posed expression labeling task. Following the format of that established posed expression task (Palermo et al., 2013, 2018), which possesses good reliability and validity, the NELT is an analogous 100-item expression labeling task with naturalistic images displaying anger, disgust, fear, happiness, or sadness (see “Method” for further details of the new naturalistic task and supplement S1 for details of the included items). Naturalistic facial expressions were sourced from the ANU Real Facial Expressions Database (ANU RealFED; ANU Emotions & Faces Lab, 2019), which contains videos depicting a variety of naturalistic contexts (e.g., reality television programs, television dramas, advertisements, game shows, talk shows, and video blogs). In our new labeling task, naïve human observers viewed static images of people’s facial expressions extracted from these clips, and selected which of five labels (anger, disgust, fear, happiness, or sadness) best described the expression shown. Importantly, labeling accuracy (percentage correct responses) was defined as agreement with the consensus label for each stimulus. In other words, the NELT measures expression *perception* with respect to the physical characteristics of the stimulus, *not* an individual’s ability to correctly identify the underlying emotion, which is typically unknowable for naturalistic stimuli. The development of the NELT addresses the clear need for a reliable naturalistic labeling task.

It is also important that the NELT be able to reveal meaningful relationships with related constructs. Therefore, across three studies, we investigated whether the NELT and the corresponding posed expression task differed in their associations with measures of empathy and general cognitive ability. These variables were carefully chosen to test the validity of the NELT against two distinct domains, which were operationalized in different ways. Cognitive and affective empathy were assessed using a self-report measure, namely, the psychometrically validated Questionnaire of Cognitive and Affective Empathy (Reniers et al., 2011). In contrast, consistent with previous investigations (e.g., Gignac & Stevens, 2024; Lin & Bates, 2022), we measured general cognitive ability with three tasks: the Concrete and Abstract Word Synonym Test (a test of verbal ability; Warrington et al., 1998), Baddeley’s Grammatical Reasoning Test (a test of processing speed; Baddeley, 1968; Hartley & Holt, 1971), and the Paper Folding Test (a test of visuospatial ability; Ekstrom et al., 1976). We used principal component analysis (PCA) to extract a general indicator of cognitive ability. The use of these different task types for empathy and general cognitive ability was important for ensuring that the ability of the NELT to reveal associations with other variables did not rely on a specific task format. We also ensured that all of our tasks were suited to studying individual differences (Goodhew & Edwards, 2019).

To test the associations of expression labeling ability with empathy and general cognitive ability, we adopted a sequential approach. We first examined the effect of general cognitive ability over and above relevant (control) demographic variables, and then added cognitive or affective empathy in separate models to see whether empathy explained any additional variance. Our decision to examine general cognitive ability prior to including empathy stems from the preexisting strength of evidence for a relationship between expression perception and general cognitive ability (Schlegel et al., 2020). Thus, our approach allowed us to isolate the effect of empathy (where evidence for a relationship with expression perception is comparatively mixed) by controlling for as many relevant variables as possible. By including general cognitive ability only after controlling for relevant demographic variables, this approach simultaneously allowed us to treat general cognitive ability as a variable of theoretical interest.

The studies reported were conducted as part of a broader research program. Data from Study 1 informed the development of the NELT and refinement of the corresponding posed task (to match the number of items retained in the NELT for Study 2). Study 1 also included the previously described measures of empathy and cognitive ability. Study 2 was aimed at investigating the psychometric properties of the NELT further via a test–retest approach, and therefore consisted of two sessions, each containing either the empathy measure or the cognitive ability tasks. Finally, Study 3 directly compared the associations of the NELT and posed expression task with empathy. Hypothesis testing directly relevant to the research questions addressed in this paper was performed only after all data collection was complete. We also used mini meta-analysis (Goh et al., 2016) to combine results across studies where possible.

## Method

### Transparency and openness

We report how we determined our sample size, all data exclusions (supplement S2), all manipulations, and all measures in the study, and we follow Journal Article Reporting Standards (Kazak, 2018). Data were analyzed using R (R Core Team, 2023). Data, information about how to access the NELT, and analysis code are available at https://osf.io/3x825/. None of the reported studies were preregistered.

### Participants

 For typically sized correlations in individual-differences research (i.e., |.20|), a sample size of 190 would be required to achieve 80% statistical power (Gignac & Szodorai, 2016). Therefore, we aimed for a sample size of at least 200 individuals per study. To cover any exclusions (detailed in supplement S2), we recruited as many additional participants as possible, given practical constraints (e.g., deadlines for student research projects). No hypothesis testing was performed until data collection was complete. Each study included a different set of participants, and we describe each sample below.

#### Study 1

A total of 386 participants completed Study 1 in 2021, with the final (i.e., post-exclusion) sample comprising 330 individuals (170 female, 155 male, 5 nonbinary) aged 18 to 56 years (*M* = 26.7; *SD* = 8.5). Participants were undergraduate students from the Australian National University (*n* = 159) who participated in the study as an educational activity, and individuals recruited via the Testable Minds online platform (https://minds.testable.org/) (*n* = 171). Testable Minds participants were reimbursed US $14 for the hour-long session. The study was approved by the Human Research Ethics Committees of the Australian National University and the University of Western Australia (UWA), and all participants gave informed consent.

#### Study 2

A total of 252 participants completed Study 2 in 2023 via Testable Minds, with the final sample comprising 219 individuals (106 female, 108 male, 2 nonbinary, 3 preferring not to say/missing) aged 19 to 67 years (*M* = 38.7; *SD* = 10.4).^2^ This study included two sessions. Participants were reimbursed US $7 for Session 1 and US $2.5 for Session 2. The study was approved by the UWA Human Research Ethics Committee, and all participants gave informed consent.

#### Study 3

A total of 303 participants completed Study 3 in 2023, with the final sample comprising 249 individuals (195 female, 52 male, 1 nonbinary, 1 preferring not to say/missing) aged 18 to 69 years (*M* = 21.5; *SD* = 6.3). Participants were undergraduate students from either UWA (*n* = 120) or Curtin University (*n* = 129) who completed the task as an educational activity and were reimbursed with partial course credit, or individuals who volunteered as friends and family of the Curtin University experimenters. The study was approved by the Human Research Ethics Committees of UWA and Curtin University, and all participants gave informed consent.

### Measures and general procedure

The measures used in each study are listed in Table 1 and described in detail below. Studies 1 and 3 were completed in a single session, while Study 2 was a test–retest study with Session 2 completed 1 week after Session 1. Participants completed the studies via the Testable online platform (https://www.testable.org) on their personal computers, with screen calibration performed at the beginning of each session. Further details of task administration and demographic questions are provided in supplement S3.

**Table 1:** Measures used in each study

	S1^a^	S2a^b^	S2b	S3^c^
Demographics	Yes	Yes		Yes
NELT	144 items	100 items	100 items	100 items
Posed expression task	144 items			100 items
Synonym test	Abstract	Abstract + concrete		
A-B test	Form A	Form A + B		
Paper Folding	Form A	Form A + B		
QCAE	Yes		Yes	Yes

NELT = Naturalistic Expression Labeling Task; QCAE = Questionnaire of Cognitive and Affective Empathy (Reniers et al., 2011)

^a^ In this study, the expression labeling tasks were counterbalanced, and the Cambridge Face Memory Test (Duchaine & Nakayama, 2006) was administered prior to the second expression labeling task

^b^ S2a refers to Session 1 of Study 2, a test–retest study; S2b refers to Session 2 of that study

^c^ Curtin University participants completed a series of other measures following the labeling tasks, including the Depression Anxiety Stress Scale-21 (Lovibond & Lovibond, 1995), Autism-Spectrum Quotient (Baron-Cohen et al., 2001), and Big Five Inventory (John et al., 1991). The results from these measures are not reported in the current paper. All participants in this study completed the NELT prior to the posed expression task

#### Expression labeling tasks

In this section, we provide details of the stimuli and procedure for both the posed expression labeling task and the NELT. We then describe the process leading to the development of the NELT and subsequent refinement of the corresponding posed task.

##### Posed expression labeling task (Palermo et al., 2013, 2018).

***Stimuli.*** Stimuli were from the Karolinska Directed Emotional Faces Database (KDEF; Lundqvist et al., 1998) and showed one of three different viewpoints (i.e., full face, left-facing three-quarter pose, and right-facing three-quarter pose). Images were of White male and female young adults displaying anger, disgust, fear, happiness, sadness, or surprise. The KDEF actors were instructed to express a given emotion strongly and clearly following a set of written instructions, to try to evoke the emotion and express it in a way that was natural to them, and to rehearse the expressions for 1 h prior to photographing (Lundqvist et al., 1998). The original 144-item posed expression labeling task with 24 images per emotion (Palermo et al., 2013, 2018) was administered in Study 1. A refined 100-item version, which omitted the surprise expressions and 20 other items (see “Test development procedure”), was administered in Study 3.

***Procedure***. Faces were presented one at a time, and participants selected the label they believed best described the expression shown (from the options “anger,” “disgust,” “fear,” “happiness,” “sadness,” and, for the 144-item task, “surprise”). Each face was presented for 400 ms, with participants given an additional 7 s to respond after the face had disappeared (median reaction time [RT] to respond was 1.6 s across Studies 1 and 3). The order of stimulus presentation was the same for each participant. To determine the order of stimulus presentation, trials were initially randomized and then adjusted such that no more than three consecutive stimuli showed the same expression. Scores for the task were calculated as percentage correct responses, with only the 100 items from the final version considered in this calculation (i.e., for Study 1, where the 144-item version was administered, we omitted the excluded 44 items when calculating percentage correct responses; scores for both versions of the task are reported in supplement S1).

##### Naturalistic Expression Labeling Task (NELT)

***Stimuli.*** Stimuli were still frames extracted from videos in the ANU RealFED (ANU Emotions & Faces Lab, 2019), a database of 1,934 YouTube video clips showing facial expressions sourced from reality television programs, television dramas, advertisements, game shows, talk shows, and video blogs. This database contains a large variety of stimuli chosen to capture the natural variation of expressions observed in real life. The images are of White male and female adults. The original 144-item task (administered in Study 1) contained 24 images for each of anger, disgust, fear, happiness, sadness, and surprise, while the refined 100-item version (administered in Studies 2 and 3) omitted the surprise expressions and 20 other items (see “Test development procedure”).

**Fig. 1:**
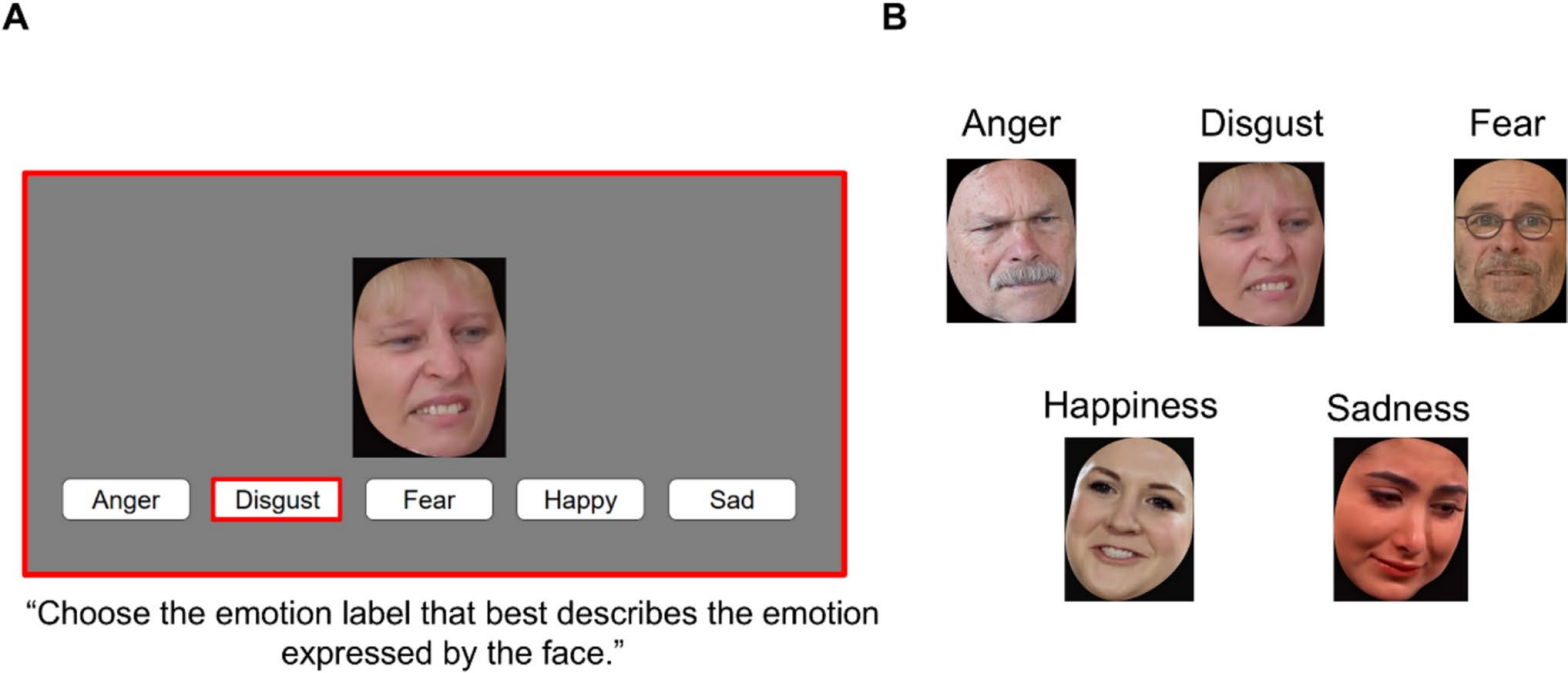
Example trial and stimuli for the Naturalistic Expression Labeling Task (NELT). **A** Example trial from the 100-item Naturalistic Expression Labeling Task (NELT), which was administered in Studies 2 and 3; trials for the posed expression labeling task were identical in format. **B** Example naturalistic stimuli for each expression in the 100-item NELT

***Procedure.*** The procedure was identical to that for the posed task, except that the naturalistic expression stimuli were presented for a longer duration (1,500 ms vs. 400 ms for posed expressions). The presentation duration was extended because pilot testing revealed the NELT to be more difficult than the posed task (consistent with the findings of Cong et al., 2025, and likely due to the inherently greater visual and affective variety in the naturalistic stimuli), and we aimed to match the mean and range of scores across the two tasks to optimize their comparability. As with the posed task, participants were given an additional 7 s following stimulus presentation to provide a response (median RT to respond was 1.8 s across all studies). Scores for the task were calculated as percentage correct responses. As with the posed task, only the final 100 items were considered in this calculation. An example trial from the 100-item NELT is shown in Fig. 1.

##### Test development procedure

We initially selected items for the 144-item NELT used in Study 1 based on unpublished data collected in 2019 for the ANU RealFED (Williams, 2019). These data were from 239 participants who viewed a random sample of 200 images from the ANU RealFED and judged what expression was being shown from the options “happiness,” “surprise,” “fear,” “sadness,” “anger,” “disgust,” or “contempt”; alternatively, participants could choose “other emotion,” “no emotion,” or “I know this person.” Participants also indicated the perceived genuineness of each expression on a scale where − 7 = *completely fake*, 0 = *don’t know*, and + 7 = *completely genuine* (minimum number per stimulus, *n* = 13) (see Dawel et al., 2017, for details of this rating task). To be selected for the 144-item NELT, each expression required a modal labeling consensus of 33.3% or above with respect to the emotion shown, and a positive average genuineness rating (i.e., 1 or above). To align with the format of the 144-item posed task reported in Palermo et al. (2013), 24 stimuli were selected for each of anger, disgust, fear, happiness, sadness, and surprise.

The 144-item NELT was administered in Study 1 of this paper, and based on data from this study and a separate sample of UWA participants who completed the 144-item naturalistic and posed tasks (combined *N* = 1,483; see supplement S1), we refined the task to a 100-item version. In refining the task, the primary goals were to maximize internal reliability and ensure the task captured a wide range of performance levels, thus enhancing its potential to reveal associations with other individual abilities and characteristics (Goodhew & Edwards, 2019). As a first step, we removed items where the modal response did not match the intended label (e.g., if a stimulus was labeled “happy,” but the most frequent response was “surprise,” we removed that item). This step was performed to ensure consensus regarding the intended label and participants’ responses. We also removed faces that were recognized by more than 5% of participants to ensure that labeling accuracy was not inordinately enhanced by familiarity (e.g., Li et al., 2019). We then followed guidance from Wilmer et al. (2012) to maximize the task’s internal reliability. For each item, we examined “alpha if item removed” values (i.e., Cronbach’s α for the task after a particular item is removed) and item correlations (i.e., the correlation between each item and performance on the remainder of the task), which indicate whether an item provides nonzero information about the ability being measured (Wilmer et al., 2012). Our approach was to first examine “alpha if item removed” values to retain stimuli that positively contributed to the internal reliability of the task and, where possible, the subtask for any given expression. We then checked that all retained items had a positive item correlation. Throughout this process, we examined the difficulty of each item (i.e., the proportion of participants who responded to the item correctly) to ensure the subtasks for each expression had a range of item difficulties. This process of balancing effects on internal reliability and score distributions was performed iteratively until we optimized both aspects of the task.

The final, 100-item version of the NELT contained 20 stimuli for each of anger, disgust, fear, happiness, and sadness. The surprise subtask in the 144-item version had poor psychometric properties that could not be improved with alterations to the task and was therefore removed. To enable comparisons across tasks, the original 144-item posed task was also refined down to a 100-item task using the same test development sample and procedure, with items showing surprise removed as a first step. Supplement S1 contains details of the individual items in the naturalistic and posed tasks.

#### ***Questionnaire of Cognitive and Affective Empathy (QCAE; ***Reniers et al., 2011***)***

The Questionnaire of Cognitive and Affective Empathy (QCAE; Reniers et al., 2011) is a 31-item scale that measures cognitive (19 items) and affective empathy (12 items) using a four-point Likert scale ranging from 1 (“strongly disagree”) to 4 (“strongly agree”). Example items are *“I can easily tell if someone else is interested or bored with what I am saying”* (cognitive empathy) and *“I am inclined to get nervous when others around me seem to be nervous”* (affective empathy). Total scores were calculated for each subscale by summation.^3^ The two-factor structure of the QCAE was verified by Reniers et al. (2011) via confirmatory factor analysis, and the questionnaire had excellent internal reliability in the present study (cognitive empathy α = .89 to .91; affective empathy α = .81 to .85).

#### Cognitive ability tasks

We administered three cognitive ability tasks: the Concrete and Abstract Word Synonym Test (Warrington et al., 1998), Baddeley’s Grammatical Reasoning Test (Baddeley, 1968; Hartley & Holt, 1971), and the Paper Folding Test (Ekstrom et al., 1976). The number of tests administered means that our battery of cognitive tasks satisfies the criteria for a “good” measure of general cognitive ability (Gignac & Bates, 2017; see Table 1).

##### **Concrete and Abstract Word Synonym Test (**Warrington et al., 1998**)**

In this test, the task is to select the synonym for a target word from two available options (e.g., for the target word”*GARRULOUS*,” options include the incorrect answer “*NOISY*” and correct answer *“TALKATIVE”).* The test has two forms (concrete words and abstract words), each comprising 25 statements. Participants have a 5-s window in which to respond on each trial, with timed-out trials scored as incorrect. Higher scores on this test (percentage correct responses) indicate greater crystalized intelligence. The test demonstrated good reliability in the present study (abstract α = .66 to .75; concrete α = .72; whole test α = .84).

##### Baddeley’s Grammatical Reasoning Test (Baddeley, 1968; Hartley & Holt, 1971)

This test presents a series of items that refer to a relation between the letters A and B—for instance, “*A comes before B: BA*.” The task is to determine whether the order of the letters following the colon is consistent with the statement by indicating either “TRUE” or “FALSE.” The test has two forms (Form A and Form B), each comprising 32 statements. For each form, participants must respond to as many statements as possible within 90 s, with incomplete trials scored as incorrect. Higher scores on this test (calculated as percentage correct responses) indicate greater cognitive processing speed. The test demonstrated good reliability in the present research (Form A α = .86 to .88; Form B α = .87; whole test α = .93).

##### Paper Folding Test (Ekstrom et al., 1976)

Each item in this test shows an image of a piece of paper being folded along a dotted section, followed by holes being punched through a particular location on the paper. The task is to determine where the holes would be located once the piece of paper has been unfolded, with six images to choose from. The test has two forms (Form A and Form B), each comprising 10 items. For each form, participants were asked to complete as many of the 10 items as possible within 3 min (Study 1) or 3.5 min (Study 2). Higher scores on this test (calculated as percentage correct responses) indicate greater visuospatial ability. The test showed acceptable reliability in the present study (Form A α = .67 to .70; Form B α = .69; whole test α = .85).

## Results

### Data analysis plan

We began by examining the internal reliability of the NELT, the relationship between performance on the NELT and that on the corresponding posed task, and bivariate associations between the NELT and other key variables (general cognitive ability, cognitive and affective empathy, prior exposure, and age). For comparison, we also report the corresponding results for the posed task. We then performed hierarchical multiple regressions to examine the unique relationships between naturalistic/posed expression labeling ability and general cognitive ability and empathy in each study. To synthesize results across studies, we followed up with fixed-effects meta-analyses on the semipartial correlations for these relationships whenever there were two or more effects addressing the same question. We opted to use semipartial correlations in the meta-analyses because they indicate the unique amount of variance in expression labeling ability explained by the predictor of interest. We generated correlation tables using the Hmisc package in R (Harrell, 2024), ran hierarchical regressions using the stats (R Core Team, 2023) and lm.beta (Behrendt, 2023) packages, and calculated semipartial correlations using the package ppcor (Kim, 2015). Meta-analyses were run using the metafor package (Viechtbauer, 2010), and the cocor package was used to statistically compare correlations (Diedenhofen & Musch, 2015). Note that for Study 2, a test–retest study, analyses involving the expression labeling tasks were completed on the averaged scores from Sessions 1 and 2.

At Step 1 of each hierarchical regression, we entered our two control variables: age and prior exposure to faces that are the same ethnicity as the test faces. These control variables were selected based on previously reported associations with expression perception (Elfenbein & Ambady, 2002; Ruffman et al., 2008). Age in years was entered as a continuous predictor. Prior exposure was a dichotomous variable, with 1 indicating a participant was White (as per the faces in the labeling tasks) and had only lived in a country with a predominantly White population before the age of 18, and 0 indicating that at least one of these conditions was not met. In Step 2, we entered our general cognitive ability variable for all studies that included the cognitive ability tasks (Study 1 and Study 2). This continuous variable was derived via PCA of the three cognitive ability tasks (half of each task for Study 1 and the full tasks for Study 2). For both studies, only one principal component had an eigenvalue greater than 1, and this component accounted for over half the variance in cognitive ability scores (53% in Study 1 and 56% in Study 2). All three cognitive ability tasks had positive loadings on this general factor (range, .66 to .81 in Study 1 and .60 to .82 in Study 2). Participants’ scores on this component were therefore used as a proxy measure of general cognitive ability; the internal consistency reliability of these scores was .55 in Study 1 and .61 in Study 2 (Armor, 1973), which may be considered acceptable for research purposes (Gignac, 2025). In the last step of each model, we entered either cognitive or affective empathy scores from the QCAE. We ran separate models for each form of empathy to obtain the change in *R*^2^ uniquely attributable to a particular type of empathy, and to avoid issues with multicollinearity (Pearson’s *r* for relationship between cognitive and affective empathy = .34 to .37). Semipartial correlations involving general cognitive ability were calculated by controlling for the demographic variables (i.e., equivalent to Step 2 of the regression models), while those involving empathy were calculated by controlling for both the demographic variables and general cognitive ability if measured (i.e., equivalent to Step 3 of the regression models).

### The NELT and posed expression labeling task: Reliable measures linked to general cognitive ability and empathy

**Fig. 2:**
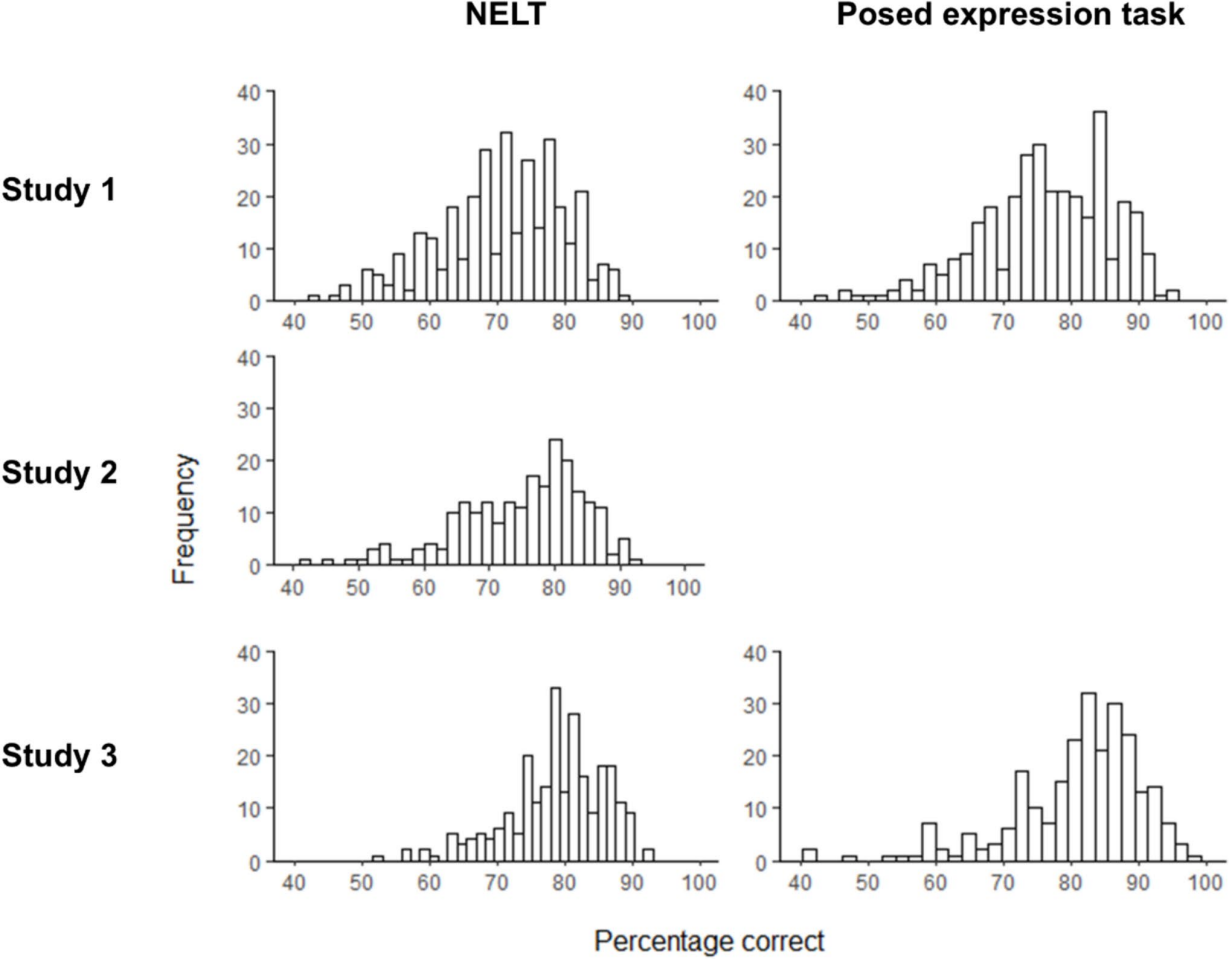
Distribution of scores on the expression labeling tasks. NELT = Naturalistic Expression Labeling Task. For Study 2, where the NELT was administered twice over two sessions, the average of the two NELT scores for each participant is plotted. The posed expression task was not administered in Study 2

Both the NELT and the corresponding posed task demonstrated good internal reliability (α for NELT, .73 to .86; for posed task, .84 and .86) and good range, with performance neither at floor nor at ceiling (Table 2; Fig. 2). The NELT also showed good test–retest reliability (Pearson’s *r* = .78, measured in Study 2), and performance was stable across time, with no significant difference between Session 1 and Session 2 scores, *t*(218) = − 1.06, *p* = .290, Cohen’s *d* = − 0.05 (see supplement S6 for additional analyses of the test–retest data). The cognitive ability tasks and cognitive and affective empathy measures similarly showed good reliability and range (Table 2). The NELT and the posed task were highly correlated (*r* = .64 in Study 1 and .60 in Study 3; *p*s < .001). The NELT was also found to be more difficult than the posed task in both Study 1, *t*(329) = − 5.55, *p* < .001, Cohen’s *d* = 0.69, and Study 3, *t*(248) = − 4.24, *p* < .001, Cohen’s *d* = 0.27 (consistent with Cong et al., 2025). Performance on both labeling tasks was consistently positively associated with prior exposure (NELT, *r* = .16 to .20, all *p*s < .01; posed, .12 in Study 1 and .14 in Study 3, *p*s < .05), but showed weaker and less consistent negative associations with age (NELT, *r* = − .10 to − .29, *p*s ranging from n.s. to < .001; posed, .03 in Study 1 and − .18 in Study 3, n.s. and *p* = .005, respectively). Cognitive ability was positively associated with age in Study 2 (*r* = .21, *p* = .002), but Study 1 showed no association between the two variables (*r* = .02, n.s.).

**Table 2:** Descriptive Statistics for Each Measure Across Three Studies

	*N*			Mean (*SD*)				Observed range				Possible range		Cronbach’s α			
	S1	S2	S3	S1	S2a^a^	S2b	S3	S1	S2a	S2b	S3	Min (chance)	Max	S1	S2a	S2b	S3
NELT^b^	330	219	249	70.8 (*9.2*)	74.7 (*9.7*)	75.2 (*10.4*)	78.9 (*7.2*)	43–89	40–92	37–95	53–93	0 (20)	100	.81	.84	.86	.73
Posed expression task	330	-	249	76.3 (*9.6*)	-	-	81.0 (*9.7*)	43-95	-	-	41–98	0 (20)	100	.84	-	-	.86
Synonym Test^c^	330	219	-	72.1 (*14.1*)	74.8 (*13.6*)	-	-	28–100	36–100	-	-	0 (50)	100	.66	.84	-	-
A-B Test^d^	330	219	-	65.4 (*18.5*)	55.0 (*17.0*)	-	-	12.5-100	14.1–98.4	-	-	0 (50)	100	.86	.93	-	-
Paper Folding^e^	330	219	-	57.2 (*23.0*)	52.4 (*22.8*)	-	-	10–100	0–100	-	-	0 (16.7)	100	.70	.85	-	-
QCAE-cognitive	324	216	248	59.9 (*8.0*)	-	57.3 (*9.2*)	61.6 (*8.6*)	34–76	-	29–76	30–76	19	76	.89	-	.91	.91
QCAE-affective	329	217	247	35.1 (*5.8*)	-	33.1 (*6.5*)	36.0 (*5.9*)	14-–47	-	14-48	20–48	12	48	.81	-	.85	.84

*NELT* Naturalistic Expression Labeling Task; *QCAE* Questionnaire of Cognitive and Affective Empathy (Reniers et al., 2011)

^a^S2a refers to Session 1 of Study 2, a test-retest study; S2b refers to Session 2 of that study

^b^For Study 1, mean accuracy on the labeling tasks was calculated for only the 100 items in the final versions of the tasks. For Studies 2 and 3, trials where participants indicated they recognized the face (see supplement S3) were not considered when calculating mean accuracy.

^c^Data for Study 1 are for the abstract word test, while data for Study 2 are for the full test

^d,e^Data for Study 1 are for Form A of the test, while data for Study 2 are for the full tests

Turning to the associations between the labeling tasks and our key individual differences variables (Table 3), both the NELT and the posed task were significantly associated with general cognitive ability (NELT, *r* = .30 [.45 disattenuated for measurement error] in Study 1 and .46 [.64 disattenuated] in Study 2; posed, *r* = .21 [.31 disattenuated] in Study 1; all *p*s < .001), while correlations between the labeling tasks and cognitive and affective empathy were less consistent (cognitive empathy, Pearson’s *r* = .08 to .18 for the NELT and .11 to .14 for the posed task; affective empathy, Pearson’s *r* = .15 to .21 for the NELT and .11 to .15 for the posed task; *p*s ranging from n.s. to < .001). See supplement S4 for further descriptive statistics (e.g., for each expression) and the full correlation table.

**Table 3:** Correlations between the expression labeling tasks and measures of empathy and general cognitive ability

Measure	Study	NELT	Posed expression task	Synonym test	A-B test	Paper Folding	Cognitive ability factor scores	QCAE-cognitive	QCAE-affective
NELT	1 2 3	– – –	.64*** (.78) – .60*** (.76)	.20*** (.27) .35*** (.42) –	.25*** (.30) .40*** (.45) –	.20*** (.27) .28*** (.33) –	.30*** (.45) .46*** (.64) –	.08 .02 .18** (.22)	.21*** (.26) .16* (.19) .15* (.19)
Posed expression task	1 2 3	.64*** (.78) – .60*** (.76)	– – –	.17**(.23) – –	.16** (.19) – –	.11* (.14) – –	.21*** (.31) – –	.11 – .14* (.16)	.15** (.18) – .11

NELT = Naturalistic Expression Labeling Task; QCAE = Questionnaire of Cognitive and Affective Empathy (Reniers et al., 2011). A dash indicates where an estimate was not available. Correlations in parentheses are those that have been disattenuated for measurement error (Spearman, 1904). For Study 1, where the 144-item labeling tasks were administered, correlations involving these tasks were calculated from the scores derived from the final 100 items. ***p < .001; **p < .01; *p < .05

### General cognitive ability predicts performance on both the NELT and the posed expression labeling task

**Fig. 3:**
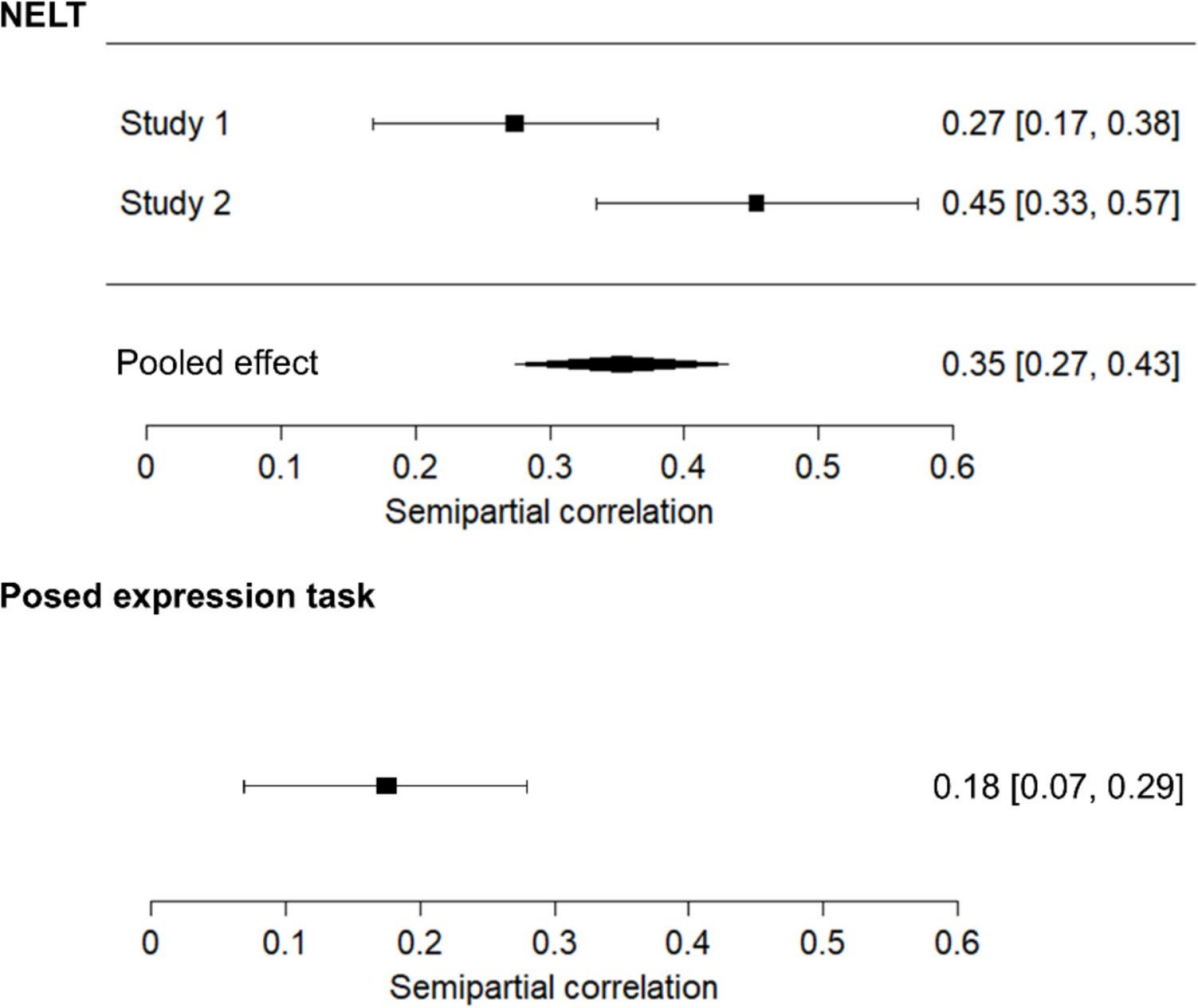
General cognitive ability and expression labeling ability. The top panel shows a forest plot for the meta-analysis on semipartial correlations between general cognitive ability and performance on the Naturalistic Expression Labeling Task (NELT). The bottom panel shows the point estimate available for the posed task (from Study 1). Bars for each study shown in the forest plot indicate 95% confidence intervals, while the length of the diamond in the top panel indicates the 95% confidence interval for the pooled effect

General cognitive ability consistently predicted performance on the NELT over and above the age and prior exposure covariates, as indicated by the significant *R*^2^ change at Step 2 in all models that included general cognitive ability (see Tables 4 and 5). Similar findings were observed for the posed task**.** For the NELT, *R*^2^-change values indicated that general cognitive ability predicted an additional 7% and 21% of variance in Studies 1 and 2, respectively (*p*s < .001).^4^ For the posed expression task, general cognitive ability predicted an additional 3% of variance in Study 1 (*p* < .001; not tested in Study 2). Importantly, for all studies, the regression coefficients for general cognitive ability were significant even when empathy scores were included in the final step (see Tables 4 and 5). A meta-analysis synthesizing the two semipartial correlations for the NELT and general cognitive ability revealed a significant overall effect of .35 (95% CI [.27, .43], *p* < .001). For comparison, the semipartial correlation for the posed task and general cognitive ability in Study 1 was .18 (95% CI [.07, .29], *p* = .002). These correlations differed significantly from one another (Fisher’s *z* = 2.28, *p* = .023), such that performance on the NELT had a larger association with general cognitive ability than did performance on the posed task.^5^ A forest plot illustrating the individual and summary effect sizes is shown in Fig. 3.

**Table 4:** Hierarchical regressions involving cognitive empathy

		Study 1					Study 2					Study 3				
		Adj.* R*^*2*^	*R*^2^ ch.^a^	Beta	Part	*p*^*b*^	Adj.* R*^*2*^	*R*^2^ ch	Beta	Part	*p*	Adj.* R*^*2*^	*R*^2^ ch	Beta	Part	*p*
A. NELT and cognitive empathy																
Step 1: Age, prior exposure		.058	.064			** < .001**	.046	.055			**.003**	.052	.060			** < .001**
Step 2: General cognitive ability		.126	.070			** < .001**	.258	.214			** < .001**	–	–			–
Step 3: Cognitive empathy		.133	.010			.055	.260	.005			.251	.073	.085			**.012**
Final model:																
	Age			. − .170	− .174	**.002**			− .215	− .232	** < .001**			− .175	− .179	**.005**
	Prior exposure			.149	.148	**.007**			.112	.122	.067			.144	.148	**.021**
	General cognitive ability			.282	.276	** < .001**			.491	.457	** < .001**			–	–	–
	Cognitive empathy			.102	.108	.055			.068	.079	.251			.158	.161	**.012**
B. Posed expression task and cognitive empathy																
Step 1: Age, prior exposure		.011	.017			.066	–	–			–	.025	.033			.025
Step 2: General cognitive ability		.042	.034			**.001**	–	–			–	–	–			–
Step 3: Cognitive empathy		.058	.019			**.012**	–	–			–	.029	.009			.142
Final model:																
	Age			.029	.029	.609			–	–	–			− .101	− .102	.112
	Prior exposure			.088	.084	.125			–	–	–			.135	.135	**.034**
	General cognitive ability			.203	.195	** < .001**			–	–	–			–	–	–
	Cognitive empathy			.140	.140	**.012**			–	–	–			.094	.094	.142

NELT = Naturalistic Expression Labeling Task; Adj. *R*^2^ = adjusted *R*^2^; *R*^2^ ch. = *R*^2^ change; Beta = standardized regression coefficient; Part = semipartial correlation. Each row shows the results of a hierarchical regression with either the NELT or the posed expression task scores as the dependent variable, with cognitive empathy scores included as a predictor in the final step. Bolded values indicate significance at the .05 level; underlined values denote significant *p* values for the *F* change resulting from the addition of key variables (general cognitive ability and cognitive empathy) to the model

^a^ The values in this column refer to the *R*^2^ change as calculated from the original (i.e., unadjusted) *R*^2^ values, because these are the values on which the corresponding *F*-change test is performed

^b^ The *p* values are for the *F*-change test at each step; in the “Final model” section, these values correspond to the test for each regression coefficient

**Table 5:** Hierarchical regressions involving affective empathy

		Study 1					Study 2						Study 3				
		Adj.* R*^*2*^	*R*^2^ ch.^a^	Beta	Part	*p*^*b*^	Adj.* R*^*2*^	*R*^2^ ch	Beta	Part	*p*		Adj.* R*^*2*^	*R*^2^ ch	Beta	Part	*p*
A. NELT and affective empathy																	
Step 1: Age, prior exposure		.060	.066			** < .001**	.043	.052			**.003**		.051	.058			** < .001**
Step 2: General cognitive ability		.128	.071			** < .001**	.254	.212			** < .001**		–	–			–
Step 3: Affective empathy		.150	.025			**.003**	.273	.022			**.011**		.058	.011			.089
Final model:																	
	Age			− .142	− .140	**.009**				− .211	− .231	** < .001**			− .164	− .163	**.010**
	Prior exposure			.132	.131	**.015**				.096	.106	.111			.154	.158	**.014**
	General cognitive ability			.273	.272	** < .001**				.482	.458	** < .001**			–	–	–
	Affective empathy			.162	.164	**.003**				.150	.173	**.011**			.108	.109	.089
B. Posed expression task and affective empathy																	
Step 1: Age, prior exposure		.011	.017			.070	–	–			–		.025	.033			**.018**
Step 2: General cognitive ability		.038	.030			**.002**	–	–			–		–	–			–
Step 3: Affective empathy		.056	.021			**.008**	–	–			–		.025	.004			.300
Final model:																	
	Age			.050	.048	.379			–	–	–				− .096	− .095	.138
	Prior exposure			.072	.068	.210			–	–	–				.144	.145	**.025**
	General cognitive ability			.179	.174	**.001**			–	–	–				–	–	–
	Affective empathy			.149	.143	**.008**			–	–	–				.067	.066	.300

NELT = Naturalistic Expression Labeling Task; Adj. *R*^2^ = adjusted *R*^2^; *R*^2^ ch. = *R*^2^ change; Beta = standardized regression coefficient; Part = semipartial correlation. Each row shows the results of a hierarchical regression with either the NELT or the posed expression task scores as the dependent variable, with affective empathy scores included as a predictor in the final step. Bolded values indicate significance at the .05 level; underlined values denote significant *p* values for the *F* change resulting from the addition of key variables (general cognitive ability and affective empathy) to the model

^a^ The values in this column refer to the *R*^2^ change as calculated from the original (i.e., unadjusted) *R*^2^ values, because these are the values on which the corresponding *F*-change test is performed

^b^ The *p* values are for the *F*-change test at each step; in the “Final model” section, these values correspond to the test for each regression coefficient

In a previous meta-analysis, Schlegel et al. (2020) observed an overall correlation of .19 between general cognitive ability and expression perception (.14 for naturalistic and .19 for posed). Our semipartial correlations cannot be directly compared with the zero-order correlations examined by Schlegel et al. (2020), given that our approach isolates the *unique* amount of variance explained by the predictor. Therefore, for comparison with Schlegel et al., we also meta-analyzed the zero-order correlations (Pearson’s *r*) available for the NELT from Studies 1 and 2. We found a summary zero-order correlation of .37 (95% CI [.29, .45], *p* < .001) for the NELT; for the posed task, the correlation (single estimate) was .21 (95% CI [.10, .31], *p* < .001). As with the semipartial correlations, performance on the NELT had a significantly stronger association with cognitive ability than did performance on the posed task, Fisher’s *z* = 2.18, *p* = .029.

### Both cognitive and affective empathy predict performance on the NELT and the posed expression labeling task

**Fig. 4:**
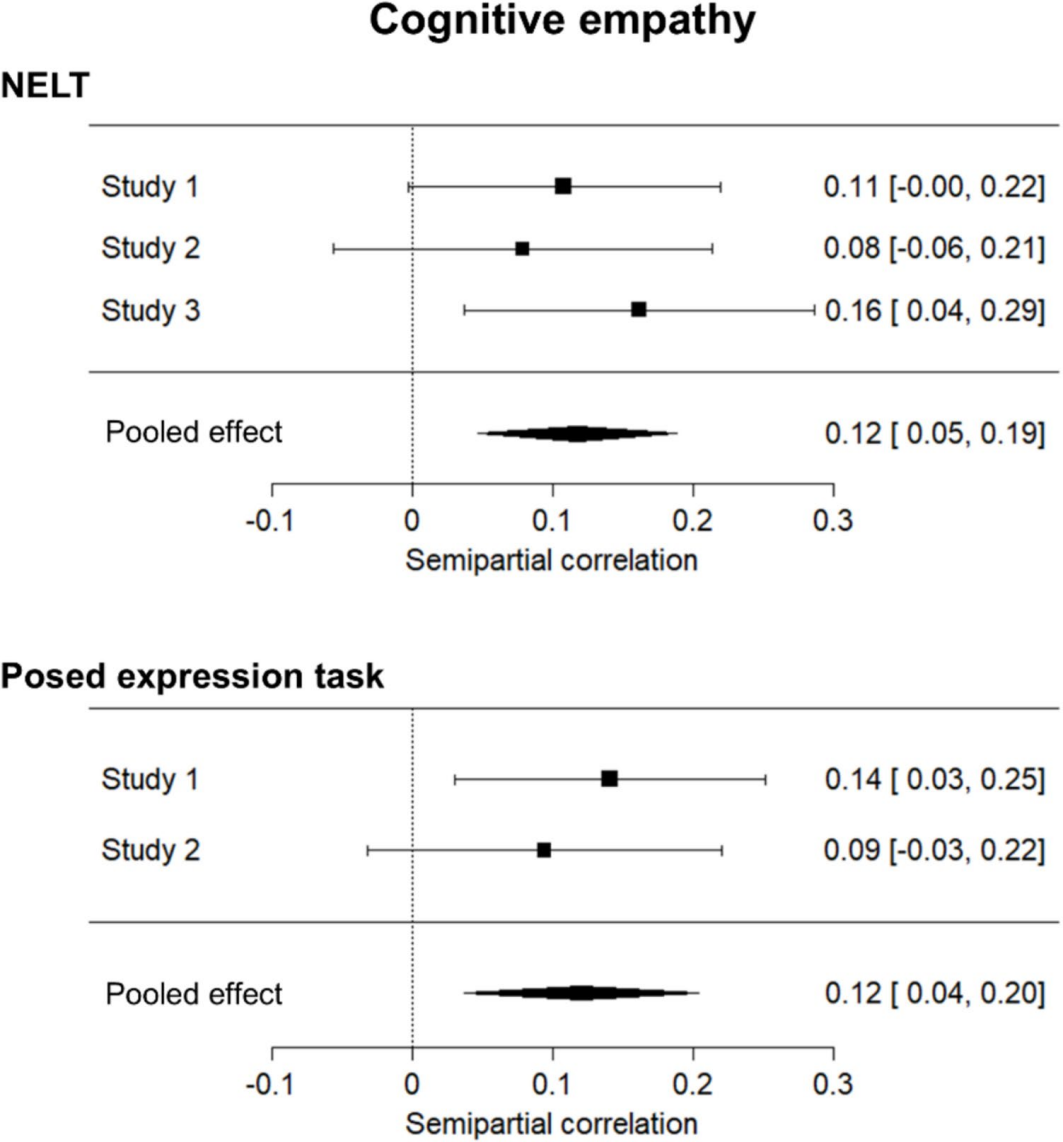
Cognitive empathy and expression labeling ability. Forest plots for the meta-analyses on semipartial correlations between cognitive empathy and performance on both the Naturalistic Expression Labeling Task (NELT; top panel) and the posed task (bottom panel). Bars for each study indicate 95% confidence intervals, while diamond length indicates the 95% confidence interval for the pooled effect

In addition to the strong association with general cognitive ability, there was also evidence of a small but robust association between performance on the NELT and cognitive empathy. Meta-analysis revealed significant effects for both the NELT (Studies 1, 2, and 3; semipartial correlation = .12, 95% CI [.05, .19],* p* = .001) and the corresponding posed task (Studies 1 and 3; semipartial correlation = .12, 95% CI [.04, .20], *p* = .005), which did not differ from one another (Fisher’s *z* = − .06, *p* = .952).^6^ Considering the individual studies, there was a significant *R*^2^ change in only two of five instances where the contribution of cognitive empathy was assessed (see Table 4), with this type of empathy accounting for 2% of additional variance in performance on both the NELT (Study 3) and the posed task (Study 1). The small size of the meta-analytic effects may help explain the inconsistent conclusions about the relationship between empathy and expression labeling across individual studies here and in the previous literature. A forest plot illustrating the individual and summary effect sizes is shown in Fig. 4.^7^

**Fig. 5:**
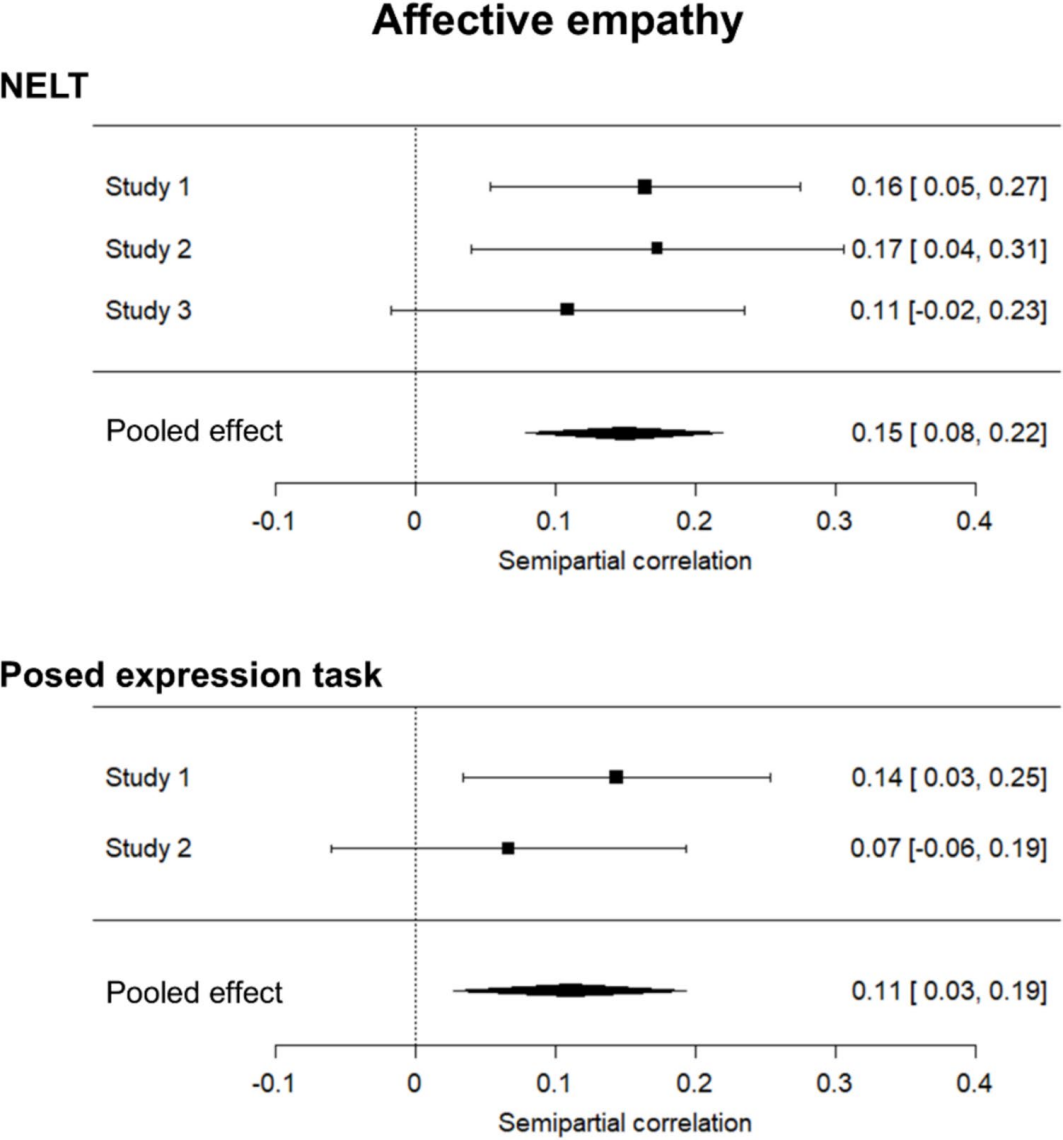
Affective empathy and expression labeling ability. Forest plots for the meta-analyses on semipartial correlations between affective empathy and both the Naturalistic Expression Labeling Task (NELT; top panel) and the posed task (bottom panel). Bars for each study indicate 95% confidence intervals, while diamond length indicates the 95% confidence interval for the pooled effect

Finally, performance on the NELT was also associated with affective empathy. Meta-analysis revealed significant effects for both the NELT (Studies 1, 2, and 3; semipartial correlation = .15, 95% CI [.08, .22], *p* < .001) and the posed task (Studies 1 and 3; semipartial correlation = .11, 95% CI [.03, .19], *p* = .009). These effects did not differ in magnitude (Fisher’s *z* = .71, *p* = .477). Results from the individual studies showed a significant *R*^2^ change in only two of four instances (Table 5), with affective empathy explaining from 2 to 3% of additional variance. Again, the small size of the meta-analytic effects could explain the inconsistency in past findings surrounding the relationship between empathy and expression labeling ability. A forest plot illustrating the individual and summary effect sizes is shown in Fig. 5.

## Discussion

Our newly developed NELT showed strong psychometric properties and revealed positive associations with measures of general cognitive ability and empathy. For general cognitive ability, associations with expression labeling ability were found to be larger for the NELT than for a well-established posed expression labeling task. The associations with cognitive and affective empathy were similar in magnitude for both the NELT and the posed expression task. A key implication of these findings is that although there are circumstances in which naturalistic and posed stimuli lead to divergent research conclusions (e.g., in schizophrenia; Davis & Gibson, 2000; LaRusso, 1978), they can also show effects in the same direction, and sometimes of similar strength. Together with the superior ecological validity of naturalistic stimuli and stronger association of the NELT with general cognitive ability, these results lead us to suggest that naturalistic stimuli should be routinely used in research examining individual differences in expression labeling ability. Given its strong psychometric properties and ease of administration, the NELT offers a valuable tool to support researchers in this transition from posed to naturalistic stimuli.

### The NELT: A psychometrically rigorous test of naturalistic expression labeling ability

Across three studies, we found that the NELT had high reliability (internal, α = .73 to .86; test–retest, Pearson’s *r* = .78) and was able to reveal associations with two important socio-cognitive variables: empathy and general cognitive ability. Combined with the strong ecological validity of the test—comprised wholly of expressions sourced from naturalistic contexts, with stimuli validated to be perceived as showing genuine emotion—as well as its easy-to-administer format, these findings show that the NELT is a valuable tool for researchers considering the use of naturalistic stimuli when examining expression labeling ability. More specifically, the observed associations with empathy and general cognitive ability demonstrate the suitability of the NELT for individual differences research aiming to uncover important relationships with other characteristics.

Our key motivations for the present research were the dominance of posed expressions in the face perception literature (Dawel et al., 2022) and the lack of psychometrically validated naturalistic tasks, making it unclear whether previous findings apply to the types of expressions we see in our everyday lives. The possibility for divergent research conclusions based on whether naturalistic or posed expressions are used is problematic for our understanding of expression perception, an ability associated with a host of positive life outcomes (e.g., Elfenbein et al., 2007; Elfenbein & Ambady, 2002) and where research on individual differences can inform strategies for improvement in those experiencing social deficits. Here, using our newly developed and validated NELT, we found positive associations with empathy and general cognitive ability that were also observed with the posed task. In addition to demonstrating the ability of the NELT to reveal important relationships, these findings illustrate a situation where testing with naturalistic and posed expressions provides converging theoretical insights into broader abilities associated with expression labeling ability.

However, while we have shown two cases where results from naturalistic and posed expressions converge, we caution against interpreting these findings as evidence that posed stimuli can be used *interchangeably* with naturalistic expressions. As outlined in our introduction, naturalistic stimuli offer several advantages over posed expressions, which are often perceived by participants as faking emotion (Dawel et al., 2017, 2025). The most critical point, however, is that people’s responses to naturalistic and posed expressions *can* differ, and therefore physical differences between the two types of expressions have real consequences for understanding human behavior (Bothe et al., 2024; Cong et al., 2025; Davis & Gibson, 2000; Dawel et al., 2019a, 2019b; Gunnery & Ruben, 2016; Krumhuber et al., 2021; LaRusso, 1978). As we discuss later, our finding that performance on the NELT demonstrated a greater association with general cognitive ability than performance on the posed task also supports the use of naturalistic stimuli. Interestingly, performance on the NELT also appeared to be more consistently associated with age and prior exposure than performance on the posed task (Tables 4 and 5), which indicates that it has more consistent associations with real-world characteristics and therefore greater ecological validity. As we have shown that it is possible to create a naturalistic task that does not compromise psychometric rigor, despite the relatively uncontrolled nature of naturalistic expressions, our recommendation is therefore that future research endeavor to use tasks consisting of naturalistic expressions—such as the NELT—when studying individual differences in expression labeling ability.

### Associations with empathy and general cognitive ability

Apart from demonstrating that the NELT can reveal important relationships with other socio-cognitive variables, our results also have broader implications for our understanding of individual differences in expression labeling ability. Our meta-analyses indicated that expression labeling ability is positively related to both cognitive and affective empathy, irrespective of whether stimuli are naturalistic or posed. However, effect sizes were small and did not reach significance in some individual studies despite relatively large sample sizes (*N*s > 200). The relatively small sizes of these effects may explain why some previous studies have not found evidence for a relationship between expression labeling ability and both types of empathy (Besel & Yuille, 2010; Gery et al., 2009; Jiang et al., 2014; Lui et al., 2016; Moret-Tatay et al., 2022; Mullins-Nelson et al., 2006; Riggio et al., 1989), while others have (Lockwood et al., 2017; Melchers et al., 2016; Olderbak & Wilhelm, 2017; Schlegel et al., 2019). These small effects highlight the need for future research to use expression labeling tasks with appropriate psychometric properties, including high reliability (Goodhew & Edwards, 2019). From a conceptual standpoint, the presence of these effects is aligned with arguments that expression perception has a role to play in empathy (e.g., Corrigan, 1997; Ochsner, 2008; Salovey & Mayer, 1990), though their small size indicates that much of the variability in empathy is related to other factors. Indeed, many other variables, such as self-reported emotion regulation (Thompson et al., 2022) and some Big Five personality traits (e.g., agreeableness; Mooradian et al., 2011), have previously been linked with empathy. To understand the relative contributions of such variables to empathy in the general population, future research should include a battery of tasks that have strong psychometric properties, suited to assessing individual differences. However, it is important to recognize that the relationship between empathy and expression perception ability might differ in specific populations. For example, even though our study shows that expression labeling ability has only a small association with empathy, potentially severe deficits in the former within some populations (e.g., in autism; Humphreys et al., 2007) could be detrimental to some aspects of empathic functioning.

Our investigation also showed a robust association between expression labeling and general cognitive ability, with evidence that this association was greater for naturalistic expression labeling than for posed labeling. This stronger association may stem from the greater nuance and variability in naturalistic expressions, which introduce a level of complexity that draws more strongly on general cognitive ability. In aligning with contemporary theories that conceptualize face processing ability (including expression perception) as a cognitive ability distinct from, but related to, general intelligence (Meyer et al., 2021; Wilhelm & Kyllonen, 2021; see also Walker et al., 2023, 2025), these findings support the use of a naturalistic labeling task. Because general cognitive ability did not entirely account for performance on the NELT, the task also captures unique variance specific to face processing, which is important for its validity as a measure of expression labeling ability. Additionally, given the evidence for a positive relationship between expression labeling and general cognitive ability from both the present study and Schlegel et al. (2020), researchers should consider controlling for general cognitive ability (where appropriate) when examining individual differences in expression labeling ability. Doing so would help isolate the unique aspects of expression labeling that are independent of general cognitive ability—such as embodied responses to facial expressions, which may facilitate perception (Krumhuber et al., 2014; Likowski et al., 2012; Niedenthal et al., 2000).

Interestingly, we observed a larger association between naturalistic expression labeling ability and general cognitive ability than did Schlegel et al. (2020) (summary zero-order correlation in the present research, .26; cf. correlation of .14 in Schlegel et al., 2020). Many of the studies included in Schlegel et al.’s meta-analysis had limited information about the expression perception tasks that were used or their psychometric properties; if the naturalistic tasks had low reliability, this could have reduced the observed associations between expression perception and general cognitive ability. Nevertheless, findings from both our study and that of Schlegel et al. suggest a clear link between expression labeling ability and general cognitive ability, supporting the expectation of convergent validity.

### Constraints on generality and future directions

The primary purpose of the NELT is to facilitate a transition to the use of naturalistic stimuli, providing a new tool for researchers and paving the way for developing further expression perception tasks that reflect real-world expressive behavior. Nevertheless, there are constraints on this measure of which users should be aware. Following the format of the posed expression task on which it was based (Palermo et al., 2013, 2018), the NELT comprises static stimuli, as opposed to dynamic stimuli. This static format enables comparisons with results obtained from preexisting posed tasks (most of which contain static stimuli), ensure a brief administration period, and also allow for testing of group differences in situations where processing speed may be a confound (e.g., older versus younger age groups). However, dynamic stimuli are more reflective of real-world expressions and may provide additional cues to the genuineness of the emotion. Therefore, development of a dynamic expression task would also be of use to researchers. Another limit on generality is that the NELT presents only White faces, matched to the ethnicity of the faces in the original posed task. To improve ecological validity, the field should consider developing more racially diverse tasks. The creation of multiracial expression labeling tasks would also dovetail with recent efforts to design racially representative tasks in other domains of emotion perception (e.g., the Multiracial Reading the Mind in the Eyes Test; Kim et al., 2024).

Regarding our individual differences findings, it is possible that a different pattern of associations for naturalistic and posed expression perception might have been revealed for response outcomes other than explicit emotion labeling. Other common measures of people’s responses to facial expressions target more implicit or automatic processes (e.g., facial electromyography [fEMG] to measure facial mimicry; Drimalla et al., 2019), which might be more sensitive to the subtleties or affective nature of naturalistic expressions. Furthermore, there is evidence that spontaneous and posed expressions may produce different patterns of neural activity (e.g., McLellan et al., 2012). Such differences could be leveraged to study individual differences in expression perception ability without explicit labeling being required, requiring the development of different types of naturalistic and posed expression tasks for comparison.

Future studies should also investigate whether behavioral, as opposed to self-report, measures of empathy reveal similar associations with expression labeling ability as those observed in the current study. Some researchers (e.g., Ickes, 1993) have criticized the use of self-report measures of empathy, arguing that individuals do not have the insight required to accurately report on such abilities. Consistent with this argument, there is evidence that associations between self-report and behavioral measures of empathy are low (Murphy & Lilienfeld, 2019). Therefore, while the QCAE is a convenient, widely used, and highly reliable measure of cognitive and affective empathy, an important future step will be to determine whether our results for empathy generalize to behavioral measures of the construct.

Finally, our studies sampled university students and the general population, excluding any participant who reported a neurological or developmental condition that might impair face processing. Therefore, there remains a need to validate the NELT with clinical and neurodiverse samples. Our findings for empathy and general cognitive ability should also be examined in terms of their applicability to other populations. For example, given evidence of differences in expression perception and empathy in schizophrenia (e.g., Bora et al., 2008), it is possible that individuals with such clinical conditions might show a different pattern of associations from those observed in the current study. Moreover, these associations might vary for naturalistic and spontaneous expressions relative to posed ones (e.g., Davis & Gibson, 2000; LaRusso, 1978).

## Conclusion

We have introduced a new, psychometrically rigorous measure of naturalistic expression labeling ability—the NELT—which shows robust associations with other socio-cognitive constructs. Evidence of associations between the NELT and measures of general cognitive ability and empathy demonstrates that testing with naturalistic stimuli can reveal robust associations with other abilities despite—or perhaps because of—the greater variation exhibited by naturalistic stimuli than their posed counterparts. Concerns that the variation in naturalistic stimuli limits detection of robust effects therefore appear to be unfounded. In view of these findings, we recommend that researchers examining expression labeling ability consider using naturalistic stimuli, as they better capture the broad repertoire of real-world expressions. However, it remains essential to ensure that tasks using naturalistic expressions demonstrate strong psychometric properties. Given its familiar format and ease of administration, the NELT offers a valuable tool to support this transition from posed to naturalistic expression stimuli.

## Data Availability

The datasets generated and analyzed in the current paper, the task materials, and all supplemental material are available in the Open Science Framework repository, https://osf.io/3x825/.

## Footnotes

1 While we specifically use naturalistic expressions in our new labeling task, our introductory review includes evidence from spontaneous expressions captured in the lab, as these also reflect natural responses to emotional events rather than deliberate posing.

2 One participant did not provide their age.

3 For both types of empathy, no score was calculated for participants with more than one missing response. If there was one missing response for either form of empathy, scores were derived by averaging the completed items.

4 It is likely that the difference between Studies 1 and 2 in the amount of variance explained by general cognitive ability is due to Study 1 including only half of each cognitive ability task, and Study 2 including the full versions of each task. That is, by using more items to measure general cognitive ability, Study 2 would have produced individual participant scores that are more reliable and thus better able to capture the underlying association between the latent constructs.

5 One possibility is that this greater association arose because we had an additional estimate for the NELT from Study 2, which included the full versions of the cognitive ability tasks (rather than half of each task, as in Study 1). The full tasks may have provided a more reliable measure of cognitive ability (see Footnote 4) and therefore increased the likelihood of observing an association between performance on the NELT and cognitive ability. Supplement S5 includes results from additional analyses that eliminate this possibility, along with the possibility that the NELT showed a stronger relationship with general cognitive ability because it was more difficult than the posed task.

6 Study 3 did not include (and thus could not control for) the cognitive ability tasks. Therefore, we also performed meta-analyses on the semipartial empathy correlations from Studies 1 and 3 *without* controlling for general cognitive ability to see whether the summary effect sizes differed. Effect sizes were almost identical; see supplement S5.

7 This forest plot shows a somewhat larger semipartial correlation between cognitive empathy and performance on the NELT for Study 3 (.16) compared to Study 1 (.11) or Study 2 (.08). The most likely explanation for such differences in empathy effect sizes is study-specific factors, such as the participant sample, or simply random variation.

## Code availability

Data analysis code is available in the Open Science Framework repository, https://osf.io/3x825/.

## Declarations

### Open practices statement

Data, task materials, and analysis code are available at https://osf.io/3x825/. None of the reported studies were preregistered.
